# Microbial Dysbiosis and Male Infertility: Understanding the Impact and Exploring Therapeutic Interventions

**DOI:** 10.3390/jpm13101491

**Published:** 2023-10-13

**Authors:** Aris Kaltsas, Athanasios Zachariou, Eleftheria Markou, Fotios Dimitriadis, Nikolaos Sofikitis, Spyridon Pournaras

**Affiliations:** 1 Department of Urology, Faculty of Medicine, School of Health Sciences, University of Ioannina, 45110 Ioannina, Greece; a.kaltsas@uoi.gr (A.K.); azachariou@uoi.gr (A.Z.); nsofikit@uoi.gr (N.S.); 2 Department of Microbiology, University Hospital of Ioannina, 45500 Ioannina, Greece; eleftheria.markou4@gmail.com; 3 Department of Urology, Faculty of Medicine, School of Health Sciences, Aristotle University of Thessaloniki, 54124 Thessaloniki, Greece; difotios@auth.gr; 4 Clinical Microbiology Laboratory, Attikon General University Hospital of Athens, 12462 Athens, Greece

**Keywords:** male infertility, microbial dysbiosis, genital microbiota, sperm dysfunction, therapeutic interventions

## Abstract

The human microbiota in the genital tract is pivotal for maintaining fertility, but its disruption can lead to male infertility. This study examines the relationship between microbial dysbiosis and male infertility, underscoring the promise of precision medicine in this field. Through a comprehensive review, this research indicates microbial signatures associated with male infertility, such as altered bacterial diversity, the dominance of pathogenic species, and imbalances in the genital microbiome. Key mechanisms linking microbial dysbiosis to infertility include inflammation, oxidative stress, and sperm structural deterioration. Emerging strategies like targeted antimicrobial therapies, probiotics, prebiotics, and fecal microbiota transplantation have shown potential in adjusting the genital microbiota to enhance male fertility. Notably, the application of precision medicine, which customizes treatments based on individual microbial profiles and specific causes of infertility, emerges as a promising approach to enhance treatment outcomes. Ultimately, microbial dysbiosis is intricately linked to male infertility, and embracing personalized treatment strategies rooted in precision medicine principles could be the way forward in addressing infertility associated with microbial factors.

## 1. Introduction

Infertility, as defined by the World Health Organization, is the inability of couples to conceive after engaging in regular sexual intercourse for over one year without the use of contraception [1]. This condition, which includes instances where a full pregnancy is not sustained for at least two consecutive natural trials, has emerged as a pressing public health issue globally. Couples grappling with infertility often face emotional, social, and financial challenges, further underscoring the need for effective interventions [2].

Recent literature presents varied findings regarding male fertility trends. Some studies have documented a decline in semen parameters [3,4,5], while others have reported stable [6,7,8] or even improved semen quality over time [9,10,11]. For instance, a comprehensive study spanning from 1973 to 2011 observed a significant decline of 50–60% in sperm counts in Western nations [12]. These varied findings underscore the multifaceted nature of male fertility and the potential influence of factors such as obesity, diet patterns, and environmental toxins [13].

The human microbiome, an intricate ecosystem comprising bacteria, viruses, fungi, and protozoa, resides both internally and externally in the human body [14]. Recent advancements in the study of the human microbiome have unveiled its significant role in various physiological processes, including nutrient absorption and immune system development. Moreover, alterations in the human microbiome have been linked to the onset, progression, and management of numerous diseases [15,16,17,18].

The microbiome’s influence extends to the male reproductive system. Historically, the absence of microbial growth in male reproductive tract samples was interpreted as an absence of microbial infection. However, contemporary research has unveiled the presence of a microbiome in the male reproductive system, including seminal fluid and urine [19].

Emerging evidence suggests a pivotal role of the microbiome in male fertility [20]. Dysbiosis, an imbalance in the microbial community, has been linked to male conditions such as oligospermia [21,22], asthenospermia [22,23,24], and teratospermia [25,26]. Furthermore, recent studies have highlighted the connection between oral and gut microbiomes and their influence on systemic health, including reproductive health [27,28,29].

Precision medicine, which emphasizes tailoring medical interventions based on individual characteristics, offer a promising avenue for addressing microbial dysbiosis and its implications for infertility [30]. By integrating genetic, environmental, and microbial factors, precision medicine aims to provide more targeted diagnostics, preventions, screening, and treatments [31,32].

This review aims to provide a comprehensive understanding of the microbial factors associated with male infertility. After detailing the mechanisms and diagnostic methods, the manuscript delves into current treatment strategies and concludes with recommendations for future research directions. By exploring the role of the microbiome in male infertility, this review seeks to pave the way for innovative treatment approaches. Harnessing the insights from precision medicine and understanding the impact of microbial factors on fertility can usher in a new era in reproductive medicine, offering hope and solutions for couples facing infertility challenges.

## 2. Microbiome and Male Reproductive Health

### 2.1. Introduction to the Human Microbiome and Its Significance

The human microbiome refers to the complex ecosystem of bacteria, viruses, fungi, and protozoa living on and in the human body [33]. This intricate assembly of microorganisms, which has co-evolved alongside humans, is pivotal to our well-being, playing a crucial role in various physiological processes [34]. Each individual’s microbiota is distinct, with specific species colonizing body sites [34,35]. For instance, the gastrointestinal tract, encompassing the mouth, esophagus, stomach, small intestine, and large intestine, teems with a diverse array of microorganisms. The oral cavity, in particular, is home to a notably diverse microbiota, with hundreds of species present [36]. The skin is populated by a rich assortment of bacteria, fungi, and viruses [37]. Meanwhile, the respiratory system, which includes the nasal cavity and lungs, boasts its own unique microbiome [38]. The urogenital tract, comprising the vagina and urinary system, also harbors distinct microbial communities [39,40].

These microbial communities are integral to maintaining homeostasis in biochemical, metabolic, and immune systems [41,42]. For instance, the gastrointestinal tract, known for its rich microbial population, is essential for digestion, nutrient absorption, and immune system development [42]. Short-chained fatty acids produced in the intestines significantly contribute to these processes [43,44]. The Human Microbiome Project, initiated in 2007, aims to consolidate genetic data from diverse human microbiomes to understand the relationship between microbiome alterations and various diseases [45,46]. This project underscores the profound influence of the microbiome on human health and its role in maintaining equilibrium [34]. Research has indicated that changes in the lung microbiome might influence the progression of conditions like chronic obstructive pulmonary disease (COPD) [34]. Additionally, interactions between the microbiota and dietary components, such as polyphenols found in plant-derived foods, can impact the gut microbiota’s composition and function, further influencing human health [42].

Factors like genetics, diet, lifestyle, and environmental exposure shape the gastrointestinal microbiome [47]. Dysbiosis may lead to metabolic issues such as irritable bowel syndrome (IBS) or allergies and reproductive disorders such as polycystic ovary syndrome (PCOS) or endometriosis [48,49]. The vaginal microbiota has also been related to preterm delivery and other reproductive issues [50,51]. The male reproductive excretory ducts also have their distinct microbiota, which can influence male reproductive health [50,52]. Semen may contain microorganisms that can be transmitted between sexual partners [52].

In conclusion, the microbiota in various body sites, including gut and reproductive tracts, profoundly impacts human health and reproductive outcomes [33,53]. Further research is essential to understand the complex interactions between human microbiota and reproductive health and potential therapeutic interventions [54,55]. With the rise of precision medicine, there is potential for interventions tailored to individual microbial profiles, offering customized therapeutic approaches to male infertility issues based on a person’s unique microbiome [56].

### 2.2. Mechanism of Action of the Microbiome Elements in Healthy Conditions

Bacteria, a significant component of the microbiome, fulfill several functions that are essential for overall well-being. They play a pivotal role in digestion by enzymatically breaking down complex carbohydrates into more easily absorbable molecules [57,58]. This activity is particularly vital within the gastrointestinal tract, where a symbiotic relationship exists between bacteria and the fermentation of dietary fibers. This fermentation results in the production of short-chain fatty acids, which serve as a crucial energy source for the host organism [57,58]. Moreover, certain bacterial strains in the gastrointestinal tract are instrumental in the biosynthesis of essential vitamins, including vitamin K and several B vitamins, vital to human health [57]. Additionally, the gut microbiota plays an indispensable role in immune system regulation, ensuring a balanced immunological response [59].

Viruses, specifically bacteriophages, also exert a profound influence on the microbiome. They can infect and lyse specific bacterial cells, thereby regulating bacterial populations and maintaining microbial balance [57]. As natural predators of bacteria, bacteriophages are pivotal in controlling bacterial population size and diversity. Furthermore, they facilitate gene transfer between bacteria, playing a significant role in bacterial adaptation and evolution [57]. This gene transfer can introduce new functionalities, influencing the overall dynamics of the microbiome.

Fungi within the microbiome contribute significantly to overall health. Some fungi assist in breaking down complex compounds, such as certain fibers, that are indigestible by bacteria [57]. By participating in this process, fungi complement the broader digestive system. Moreover, by occupying specific ecological niches within the body, fungi can prevent the overgrowth of harmful pathogens through competitive inhibition, ensuring a balanced microbial ecosystem and thwarting the establishment of potentially harmful bacteria [57].

Protozoa, another component of the microbiome, play a role in maintaining overall health. Some protozoa in the gastrointestinal tract aid in digestion by breaking down specific compounds, enhancing nutrient absorption [57]. Additionally, the presence of protozoa can stimulate the immune system, bolstering its ability to recognize and combat foreign pathogens [57]. This immunological stimulation is vital for maintaining robust immune responses and protecting the body against various diseases.

In a state of good health, these microorganisms work in harmony, ensuring the optimal functioning of various physiological processes. They are integral to numerous physiological activities, including digestion, nutrient absorption, immune system regulation, and the prevention of pathogenic overgrowth. The balance and diversity of these microbial communities are paramount for health maintenance and disease prevention.

### 2.3. Microbiota in the Male Reproductive System

The male reproductive system, once perceived as largely sterile, is now recognized as a complex mosaic of microbial communities. The male genital tract, including regions like the urethra and the coronal sulcus, harbors distinct microbial communities [60]. Predominant bacterial genera in the male reproductive tract include *Corynebacterium*, *Streptococcus*, and *Staphylococcus* [61,62]. The microbial composition within this tract exhibits individual variations [61], influenced by factors such as sexual behavior, hygiene practices, and the presence of sexually transmitted infections (STIs) [63].

A balanced and diverse microbiota within the male genital system is essential for optimal reproductive health [61]. Bacteria like *Escherichia coli* and *Ureaplasma urealyticum* have been associated with chronic prostatitis, leading to inflammation and potential damage to the male reproductive system [64]. Furthermore, the microbial composition within the male urinary tract can influence susceptibility to STIs and the risk of transmission to sexual partners [60]. Dysbiosis in this region can be a contributing factor to various reproductive challenges [50,62].

Delving deeper, the testes, traditionally viewed as devoid of microbes, have been revealed to contain a low-abundance microbiota. The epididymis, a duct where sperm mature, also houses specific bacterial communities, the significance of which is still under exploration. The vas deferens, though less studied, might contain microbes influencing its function. Seminal vesicles and the prostate, glands vital for seminal fluid production, have their microbiota, which can impact the fluid’s composition and health. The urethra, connecting to the external environment, boasts a diverse microbiota influencing its health. Semen, beyond carrying sperm, also transports microbial communities that can affect sperm health and fertility. Lastly, the penile skin, including the glans and shaft, has its microbiota, influenced by factors like circumcision [61].

In essence, recognizing and understanding this intricate microbial landscape within the male reproductive system offers potential for therapeutic interventions and improved reproductive outcomes. The communities play pivotal roles in reproductive health, and their imbalances can lead to conditions like infertility.

## 3. Microbial Dysbiosis and Male Infertility

### 3.1. Definition and Characterization of Microbial Dysbiosis

Microbial dysbiosis refers to a disturbance in the structure and function of microbial populations within the human microbiota. It is marked by a shift from the typical or optimal microbial composition, leading the changes in the metabolic functions and distribution of the microbiota’s constituents [65]. The human microbiota’s composition has been linked to various health outcomes [66,67]. Dysbiosis in the male reproductive tract microbiota is considered a factor in male infertility. Recognizing individual microbial imbalances is crucial for precision medicine, enabling tailored interventions to address root causes of male infertility [68].

Oxidative stress (OS) significantly influences male infertility development. Elevated OS levels or DNA-damaged sperm increase infertility risk [69]. Dysbiosis of the male genital tract microbiota is associated with conditions like prostatitis, urethritis, and infertility [70]. Specific bacterial species such as *Escherichia coli* and *Ureaplasma urealyticum* are linked to chronic prostatitis and inflammation in the male reproductive system [71].

Dysbiosis in the male reproductive tract is complex, varying across diseases and individuals. It often involves a decrease in microbial diversity and an increase in facultative anaerobic species [65]. Such dysbiosis can lead to detrimental effects on the male reproductive system, impacting sperm quality and functionality [72]. Infections from specific microorganisms, like *Ureaplasma urealyticum,* are associated with male infertility [72].

**Figure 1:**
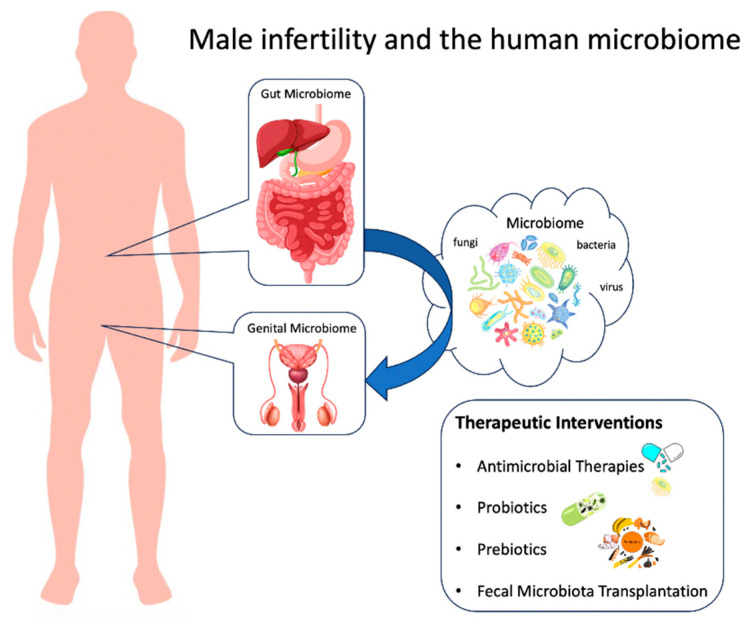
Locations, components, and potential intervention of the male microbiome.

The dysbiosis concept, especially concerning the gastrointestinal tract, is still under development. It sometimes lacks clarity and a strong scientific basis [73,74]. Yet, it remains essential in understanding the role of microbiota in health and disease [75]. Figure 1 presents an overview of the locations and components of the male microbiome, along with potential interventions. 

In summary, microbial dysbiosis in the male reproductive tract correlates with male infertility. Specific bacterial species and imbalances in the male genital tract microbiota are linked to conditions, like prostatitis, inflammation, and oxidative stress, affecting sperm quality and fertility. Further research is essential to understand the mechanisms of dysbiosis in male infertility and potential therapeutic interventions.

### 3.2. Evidence Linking Microbial Dysbiosis to Male Infertility

1. Altered bacterial diversity and richness: A hallmark of dysbiosis in the male reproductive tract microbiota is reduced microbial diversity and richness [65]. Such a decline can disrupt microbial community balance, affecting male infertility [76].
2. Proliferation of pathogenic species: Dysbiosis can lead to an overgrowth of pathogenic species in the male genital tract. Bacteria like *Escherichia coli* and *Ureaplasma urealyticum* are associated with chronic prostatitis and inflammation [77]. These species can induce OS, inflammation, and damage to the male reproductive system, impacting sperm quality and fertility [69].
3. Genital microbiome disruptions: Changes in the composition and function of the male genital tract’s microbiota are linked to male infertility. Such disruptions can cause inflammation, oxidative stress, and sperm cell damage [69].

Research supports the association between microbial dysbiosis and male infertility, highlighting changes in bacterial diversity, pathogenic species proliferation, and genital microbiome imbalances. Identifying these imbalances can guide precision medicine approaches, offering tailored treatments for male infertility [32].

### 3.3. The Impact of Gut Microbial Dysbiosis on Male Infertility

Research on the microbiome primarily centers on the gastrointestinal tract, which houses a significant portion of microbial organisms. Recent studies have drawn attention to the correlation between gut microbiota dysbiosis and male infertility, suggesting its potential significance in clinical practice [78,79,80].

Ding et al. [78] found a marked reduction in sperm concentration and motility in mice subjected to a high-fat diet. These mice also showed a decline in *Bacteroidetes* and *Verrucomicrobia*, with an increase in *Firmicutes* and *Proteobacteria* in their gut microbiota. In another study, Zhang et al. [81] noted a significant rise in sperm concentration and sperm motility after transferring fecal microbiota from alginate oligosaccharide-treated mice to busulfan-treated mice. This change was associated with an increase in “beneficial” bacterial populations, specifically *Bacteroidales* and *Bifidobacteriales* [81]. Zhao et al. [79] highlighted that alginate oligosaccharides could potentially counteract the spermatogenesis impairment caused by busulfan in mice. This effect was linked to an increase in beneficial bacteria like *Bacteroidales* and *Lactobacillaceae* and a decrease in harmful bacteria, notably *Desulfovibrionaceae* [79].

While specific research on the relationship between gut microbiota and male infertility is limited, existing studies provide valuable insights. These insights emphasize the need for further exploration into manipulating gut microbiota in infertile males. Understanding and addressing gut microbiota imbalances in infertile males can lead to innovative, tailored treatments targeting the root causes of infertility [20].

### 3.4. Microbial Dysbiosis in the Reproductive System and Its Association with Male Infertility

Microorganisms play a pivotal role in male fertility. The male reproductive tract microbiome, with its diverse bacterial composition, can influence various aspects of reproductive health [61]. For instance, the interactions of the male microbiome with the immune system can impact reproductive health, with dysbiosis exacerbating reproductive challenges [33]. Metabolites produced by the male microbiome can influence fertility, potentially affecting the reproductive system either immediately or over time. Such microbial imbalances can also alter sperm quality and other parameters [82]. The “seminovaginal microbiota”, transmitted between partners during sexual activity, can influence couple fertility. Comprehensive fertility assessments should consider the microbiota of both partners [83].

A myriad of factors, including STIs, immunological interactions, metabolic processes, and microbial dysbiosis can influence male fertility [61]. Understanding the intricate interactions between the male microbiota and fertility is imperative for developing effective therapeutic strategies. Precision medicine, which its focus on individualized care, can facilitate the development of tailored fertility treatment plans by identifying specific microbial imbalances, allowing for targeted interventions to enhance fertility outcomes [84].

While there has been extensive research on microbiota across various anatomical regions, the study of microbiota in the male reproductive system remains relatively limited. Historically, research primarily focused on identifying established pathogens through conventional culture-dependent techniques, microscopic examination, and targeted polymerase chain reaction (PCR) amplification. However, with the advent of next-generation sequencing, there has been a shift toward identifying the complex microbiota in the male reproductive system, offering a clearer understanding of its relationship with male infertility.

It is well-documented that disturbances in the microbiota of the female reproductive tract can lead to various reproductive disorders [85,86,87]. Yet, comprehensive research on the male reproductive tract microbiota has been lacking. Historically, the absence of microbial growth in tests on samples from the male reproductive tract, termed “culture-negative”, was seen as an indication of the absence of bacterial infection. This perspective led to a limited understanding of the microbiota composition of the male reproductive tract. However, emerging evidence suggests the presence of a microbiota in the reproductive tract and associated body fluids, such as seminal fluid and urine [88].

### 3.5. Dysbiosis in Specific Regions of the Male Reproductive System

#### 3.5.1. Rete Testis, Efferent Ducts, and Epididymis 

Research on the testicular microbiome is scarce. A pivotal study by Alfano et al. [89] provided initial evidence linking male infertility to changes in the testicular microbiome. Infertile men showed a lack of *Bacteroidetes* and *Proteobacteria* in the testis. Results from microsurgical testicular sperm extraction tests that yielded no sperm indicated changes in the abundance of *Firmicutes* and *Clostridium*, a complete absence of *Peptoniphilus asaccharolyticus*, and an elevation in *Actinobacteria*.

#### 3.5.2. Deferent Duct, Seminal Vesicles, and Prostate

Lundy et al. [80] observed that post-vasectomy, there was a decrease in the presence of *Collinsella* (phylum *Actinobacteria*) and *Staphylococcus* (phylum *Firmicutes*) in semen. This observation indirectly points towards a potential link between testicular microbiota and male infertility.

#### 3.5.3. Urethra and Coronal Sulcus

The male genital tract, encompassing regions such as the urethra and the coronal sulcus, harbors distinct microbial communities [60]. Predominant bacterial genera in the male reproductive tract include *Corynebacterium*, *Streptococcus*, and *Staphylococcus* [61,62].

#### 3.5.4. Semen

Studies focusing on male infertility have primarily analyzed semen samples. These studies have consistently found variations in the microbiota composition in the semen of infertile males. Notably, there is an increased prevalence of *Prevotella* and *Staphylococcus* and a decreased presence of *Lactobacillus* and *Pseudomonas* [76,90,91]. Baud et al. [91] and Farahani et al. [76] further highlighted a negative correlation between the prevalence of *Prevotella* and sperm motility. In contrast, a direct correlation was observed between a decreased abundance of *Lactobacillus* and abnormal sperm morphology.

Comparing samples from fertile and infertile individuals, rectum samples from infertile men showed variations in the abundance of *Anaerococcus*, while displaying an elevated abundance of *Lachnospiraceae*, *Collinsella*, and *Coprococcus*. Conversely, urine samples obtained from infertile men demonstrated an increased presence of *Anaerococcus*. Furthermore, semen samples from infertile men exhibited a decreased presence of *Collinsella* and an increased presence of *Aerococcus* [80]. Subsequent research revealed a statistically significant inverse relationship between the abundance of *Aerococcus* and both leukocytospermia and semen viscosity. Additionally, a statistically significant negative correlation was observed between the abundance of *Prevotella* and semen concentration. Conversely, a statistically significant positive correlation was found between the abundance of *Pseudomonas* and sperm count, while exhibiting an inverse proportionality with the pH of semen [80]. To solidify the findings from these studies, more extensive, longitudinal research across multiple institutions is essential.

## 4. Mechanisms Linking Microbial Dysbiosis and Male Infertility

Male infertility is often linked with microbial dysbiosis, which is an imbalance in the composition and function of the microbiota. This imbalance is believed to influence infertility through various mechanisms. This section delves into three primary mechanisms connecting microbial dysbiosis and male infertility: inflammation and immune response, the effect of OS on sperm quality, and the implications of impaired sperm function and motility.

1. Inflammation and immune response: A significant mechanism linking microbial dysbiosis to male infertility is the activation of inflammation and immune response. For instance, the oral microbiota, known for its dynamic and polymicrobial nature, can directly lead to diseases like dental caries and periodontitis [92]. These conditions manifest an inflammatory response triggered by the interaction between the microbiota and host factors, such as inflammation and dietary sugars [92]. In the context of male infertility, inflammation and immune response can negatively influence sperm function and overall fertility.

The generation of ROS during inflammation can induce OS [93], which is known to adversely affect fertility by compromising sperm plasma membrane fluidity and DNA integrity, leading to reduced sperm counts and impaired sperm function [94].

2. OS and its impact on sperm quality: OS, an imbalance between the ROS production and the body’s antioxidant defenses, is associated with male infertility [93]. While ROS are essential for reproduction, their overproduction can harm sperm DNA, impair sperm motility, and increase susceptibility to genetic anomalies [93]. OS can alter sperm morphology and reduce sperm concentration, affecting overall semen parameters [95]. Mechanisms through which OS affects sperm quality include lipid peroxidation, DNA damage, and compromised mitochondrial function [96].

Mitochondrial function is crucial for sperm motility, as mitochondria supply the energy required for sperm movement [96]. The motility of sperm is heavily reliant on the functionality of mitochondria, as these organelles play a crucial role in supplying the necessary energy for sperm locomotion [96]. Impaired mitochondrial function can reduce adenosine triphosphate (ATP) production, leading to decreased sperm motility and fertility [96].

3. Impaired sperm function and motility: Dysfunction in sperm movement and function are common in male infertility cases. The gut microbiota composition, influenced by factors like diet and the immune system, can affect sperm function and motility [97]. Dysbiosis in the gut microbiota, marked by reduced microbial diversity and the growth of specific bacterial taxa, has been linked to compromised sperm function and motility [97]. Factors such as oxidative stress, bacteriophage induction, and bacterial toxin release can initiate this dysbiosis [97].

It is also worth noting that pathogenic bacteria can negatively impact sperm function and motility [98]. Bacterial infections in the male reproductive tract can induce inflammation and oxidative stress, affecting sperm quality and overall fertility [8]. The presence of intracellular bacteria within the male reproductive tract can trigger an immune response, interfering with sperm function and leading to fertility issues [98].

In summary, microbial dysbiosis can negatively influence male fertility through various mechanisms, including inflammation, oxidative stress, and impaired sperm function and motility. Understanding these mechanisms is crucial for the advancement of precision medicine, especially as findings reveal altered microbial compositions in conditions like non-obstructive azoospermia (NOA) [99]. By pinpointing specific microbial imbalances and their effects on male fertility, precision medicine can design interventions to restore microbial balance and address the root causes of infertility. This tailored approach, considering each patient’s unique microbial and genetic factors, represents the future of reproductive medicine. As research continues to unravel the complexities of microbial dysbiosis and its impact on male fertility, precision medicine remains at the forefront, offering targeted and personalized solutions for couples facing infertility challenges [19].

## 5. Diagnostic Approaches for Assessing Microbial Dysbiosis in Male Infertility

### 5.1. Current Methods and Limitations

To evaluate microbial dysbiosis in male infertility, current methodologies primarily focus on analyzing microbiota composition and diversity using techniques such as culture-based techniques, PCR, quantitative PCR (qPCR), and next-generation sequencing (NGS) [28,76,100,101,102,103,104,105].

Culture-based methods involve isolating and cultivating bacteria from clinical samples, followed by identification using biochemical tests or DNA sequencing. However, these methods might not capture the full spectrum of microbial communities, often showing a bias towards culturable bacteria [100,101].

Molecular techniques, including PCR and qPCR, allow for the identification and quantification of specific microbial taxa or genes associated with dysbiosis. They provide a broader view of microbiota composition and can identify low-abundance or non-culturable bacteria [28,76,100,102,103,104,105]. However, their specificity can sometimes limit the full coverage of microbial diversity and they might not provide insights into the functional capabilities of the microbiota [76,102].

NGS technologies, utilizing 16S rRNA gene sequencing and metagenomic sequencing, have revolutionized microbial community research. They can detect and characterize both culturable and non-culturable bacteria, shedding light on functional pathways and interactions within the microbiota [76,102,103,104,105]. Yet, they come with challenges, including high costs, complex data analysis, and the need for specialized bioinformatics knowledge [76,102,103,104,105].

Despite advancements in microbial analysis techniques, challenges persist. The lack of standardized protocols and reference databases can lead to outcome variations, making comparisons across research difficult [76,102,103,104,105]. Moreover, understanding microbial dysbiosis in male infertility is challenging due to the complex and dynamic nature of the microbiota and the lack of clear microbial indicators for infertility [76,102,103,104,105].

### 5.2. Advances in Molecular Techniques for Microbiota Analysis

Recent advancements in molecular techniques have enhanced the ability to study microbiota and assess microbial dysbiosis in male infertility.

#### 5.2.1. Next-Generation Sequencing

NGS, through technologies like 16S rRNA gene sequencing and metagenomic sequencing, offers efficient DNA or RNA sequencing. This allows for a comprehensive assessment of microbial community composition and potential functionality [76,102,103,104,105].

#### 5.2.2. Metatranscriptomics

This technique examines microbial gene expression within a community, providing insights into the functional activities of the microbiota and identifying key metabolic pathways potentially disrupted in male infertility [102].

#### 5.2.3. Metaproteomics

Focusing on analyzing proteins expressed by the microbial community, metaproteomics can identify and quantify these proteins, offering insights into the functional behaviors of the microbiota and potential links to microbial dysbiosis in male infertility [106].

#### 5.2.4. Shotgun Metagenomics

This comprehensive sequencing of a microbial community’s entire DNA offers insights into the genetic capabilities of microorganisms and can detect specific genes or pathways associated with dysbiosis [102].

#### 5.2.5. Long-Read Sequencing

Technologies like PacBio and Oxford Nanopore sequencing produce extended DNA reads, resolving complex microbial communities and enhancing taxonomic and functional assignments [107].

### 5.3. Biomarkers for Identifying Microbial Dysbiosis in Male Infertility

Identifying reliable biomarkers for the assessment of microbial dysbiosis in male infertility is crucial for accurate diagnosis and targeted therapeutic interventions. Biomarkers can provide objective and quantifiable indicators of dysbiosis, aiding clinical decision-making. Several important biomarkers can be identified, namely:

#### 5.3.1. Seminal Oxidative Stress Markers 

OS markers, including ROS concentrations, lipid peroxidation, and antioxidant enzyme function, can provide insights into the oxidative state of seminal fluid and its association with dysbiosis [103,108,109].

#### 5.3.2. Seminal Inflammatory Markers 

Markers related to inflammation, such as cytokines, chemokines, and immune cell populations, can be quantified in seminal fluid to assess inflammation and its link to dysbiosis [109].

#### 5.3.3. Microbial Biomarkers 

Specific microbial taxa or genes can serve as indicators of dysbiosis in male infertility. Molecular techniques, like 16S rRNA gene sequencing, can help distinguish between healthy and dysbiotic microbiota [76,103].

#### 5.3.4. Metabolomic Biomarkers 

Metabolomic profiling of seminal fluid can reveal metabolic alterations linked to dysbiosis. Specific metabolites or metabolic pathways can be quantified to assess the metabolic state and its association with dysbiosis [102].

#### 5.3.5. Epigenetic Biomarkers 

Epigenetic changes, especially DNA methylation, play a role in male infertility. Microbial dysbiosis can influence these epigenetic modifications, and assessing changes linked to dysbiosis can be done by measuring epigenetic biomarkers in sperm DNA [110].

In conclusion, while current approaches for assessing microbial dysbiosis in male infertility have made significant strides, challenges remain. Molecular techniques and the identification of specific biomarkers align with precision medicine principles, aiming to provide individualized treatments based on each patient’s unique microbial and genetic factors. As the field continues to evolve, integrating these diagnostic tools with the broader precision medicine framework will be essential for delivering optimal care to patients facing infertility challenges [56,111].

## 6. Therapeutic Interventions for Modulating the Genital Microbiota

### 6.1. Targeted Antimicrobial Therapies

Addressing male infertility often necessitates treating underlying microbial infections that disrupt the genital microbiota balance. While targeted antimicrobial therapies present a potential solution, their application should be judicious to avoid exacerbating genital microbiota imbalances [35,70,94,95]. Interventions might include therapies such as probiotics, prebiotics, and synbiotics, which aim to rebalance the microbiota and enhance reproductive outcomes. Additionally, precision medicine might leverage molecular biomarkers and inflammatory mediators to modulate the immune response and bolster reproductive health [112].

Antibiotics, tailored to specific infections and their susceptibilities, are commonly employed to counter bacterial infections and diminish bacterial prevalence in the genital microbiota [35,70,94,95]. Yet, indiscriminate antibiotic use can perturb the commensal microbiota, potentially inducing further dysbiosis [35,70,94,95]. Antibiotics like quinolones, trimethoprim, tetracyclines, macrolides, and β-lactam have demonstrated efficacy in restoring semen parameters in infertile males with infections, leading to enhanced sperm motility and fertility outcomes [113,114].

Antifungal agents, such as azoles, counteract *Candida* species overgrowth in the genital microbiome [115]. Additionally, antiviral medications address viral infections like herpes simplex virus (HSV) and the human papillomavirus (HPV), which are associated with male infertility [116,117].

However, potential side effects warrant consideration. Some antibiotics might exhibit testicular or sperm toxicity, as suggested by rodent studies [118]. Antiviral treatment for hepatitis C virus (HCV) has been linked to adverse semen parameters in infertile males [119,120,121].

Given these considerations, targeted antimicrobial therapies should be judiciously employed, emphasizing the importance of microbiome balance and potentially integrating probiotic supplements or dietary modifications [35,70,94,95].

### 6.2. Probiotics and Their Potential Benefits

Probiotics, beneficial live bacteria consumed in adequate amounts, have emerged as potential agents to promote health and address male infertility. By fostering beneficial bacteria and suppressing harmful microorganisms, they can help reestablish a balanced genital microbiome [122,123].

Oral probiotics, such as *Lactobacillus* and *Bifidobacterium* species, can colonize the gut and indirectly influence the genital microbiota. They compete with pathogenic bacteria for resources, modulate the immune response, and produce antimicrobial compounds, potentially improving semen parameters [124,125,126,127,128,129].

Topical probiotics, especially *Lactobacillus* species, can be directly administered to the vaginal tract, aiding in reestablishing a healthy microbial balance. These probiotics are able to adhere to the vaginal epithelium, produce lactic acid to maintain an acidic pH, and inhibit the growth of harmful bacteria. This local administration could potentially be useful in improving male fertility in cases of vaginal disorders, as suggested by research that showed the protective effect of certain *Lactobacillus* strains on sperm in the presence of such conditions [130].

The efficacy of probiotics in male infertility might hinge on specific strains, dosage, and treatment duration. The beneficial impact of probiotics on male fertility and reproductive hormones has been indicated in both human and animal studies. Notably, improvements were observed in roosters, male mouse models, and zebrafish, along with hormonal changes in humans [125,126,127,128,129].

However, despite the promising results, it is crucial to remember that more research is needed to fully comprehend these effects and to determine the most effective probiotic strains, dosages, and treatment plans for various forms of male infertility. The potential role of probiotics in enhancing male fertility underscores the need for continued research in this intriguing area.

### 6.3. Prebiotics and Their Role in Restoring Microbial Balance

Prebiotics, non-digestible dietary fibers that foster beneficial bacterial growth, play a pivotal role in restoring microbial equilibrium. They support the colonization and metabolic activity of probiotic bacteria by acting as a substrate for their development [131,132,133]. Found in foods like whole grains, legumes, fruits, and vegetables, prebiotics are fermented by helpful bacteria in the upper gastrointestinal tract [7,8,9]. Short-chain fatty acids (SCFAs), including butyrate, acetate, and propionate, are created during this fermentation process and serve to maintain a balanced microbiota in the genitals and stomach [131,132,133].

By promoting beneficial bacterial growth, prebiotics can modulate the immune response, inhibit harmful bacterial proliferation, and increase antimicrobial compound production [131,132,133]. They might enhance sperm quality, reduce oxidative stress, and modulate the immune response in male infertility contexts [131,132,133]. To identify the precise prebiotic formulations, doses, and durations of therapy that are most successful in reestablishing a healthy microbial balance in male infertility, more study is required.

### 6.4. Fecal Microbiota Transplantation and Its Implications

Fecal microbiota transplantation (FMT) involves transferring fecal material from a healthy donor to a recipient, aiming to restore a balanced gut and potentially genital microbiota [35,95,112,115].

A disorder known as *Clostridioides difficile* infection (CDI), which is characterized by a dysbiosis of the gut microbiota, is predominantly treated with FMT [134]. A diversified and balanced microbial population may be restored with the transplantation of feces from a healthy donor, curing CDI symptoms [134].

FMT has attracted interest recently as a possible therapy for other ailments linked to microbial dysbiosis, such as certain gastrointestinal problems, metabolic disorders, and even diseases of the female reproductive system [35,95,115]. By altering the vaginal and intestinal microbiota, lowering inflammation, and enhancing overall reproductive health, FMT may have an impact on male infertility [35,95,115].

However, FMT remains an experimental treatment for conditions other than CDI; thus further research is needed to determine if it is safe and effective for treating male infertility [35,95,115]. For the safe and efficient use of FMT in male infertility, adequate donor selection, pathogen screening, and standardization of FMT procedures are crucial factors [35,95,115].

In conclusion, therapeutic strategies like targeted antimicrobial treatments, probiotics, prebiotics, and FMT offer potential avenues for modulating the genital microbiota in male infertility contexts. These interventions aim to enhance sperm quality, reduce OS, and regulate immune responses. However, further research is imperative to refine treatment regimens, ascertain long-term outcomes, and ensure the safety and efficacy of these therapies in clinical settings.

Aligning these therapeutic interventions with precision medicine principles offers a promising path forward. By understanding individual microbial imbalances and tailoring treatments accordingly, clinicians can provide more targeted interventions, enhancing treatment efficacy and minimizing potential side effects. As the male infertility treatment landscape evolves, integrating these therapeutic strategies within the broader precision medicine framework will be crucial for delivering optimal, individualized patient care [56].

## 7. Challenges and Future Perspectives

The exploration of the microbiome’s association with male infertility, while comprehensive, still presents significant limitations and offers ample opportunities for further research. While numerous randomized controlled trials have been included, the strength of the research evidence is compromised due to insufficient details on randomization methods, the frequent lack of adequate controls, and the dearth of double-blind clinical studies. Factors such as age, body mass index, lifestyle habits, and medication use can influence both the microbiome and reproductive parameters, adding layers of complexity to the research [135].

This review highlights challenges such as small sample sizes, diverse study designs, variability in antimicrobial or probiotics administration, and the limitations of 16S rRNA technology in sequencing. A significant number of studies have not delved into the impact of the microbiome on clinical fertility outcomes in infertile males. Therefore, there is a pressing need for large-scale prospective studies to ascertain the microbiome’s role as a causative factor in male infertility [76].

For microbial-based therapies for male infertility to be effectively implemented, a myriad of factors must be considered. This includes accurate diagnosis of microbial dysbiosis, selection of suitable antimicrobial agents, probiotics, or prebiotics, and considering potential interactions with the host’s immune system. Furthermore, optimizing the route of administration and treatment duration is essential for ensuring therapeutic efficacy. Long-term monitoring and follow-up are also pivotal for evaluating the success and safety of microbial-based interventions and for making necessary treatment adjustments.

Implementing microbial-based interventions in male infertility introduces ethical and safety challenges, encompassing informed consent, protection of personal health data and transparency in treatment utilization. The potential risks associated with antimicrobial agents, probiotics, or prebiotics necessitate thorough safety and efficacy evaluations through rigorous preclinical and clinical studies before broad implementation [136].

The horizon holds promise for personalized medicine in the realm of male infertility management. Personalized medicine seeks to tailor medical interventions based on individual characteristics, encompassing genetic, environmental, and microbial factors. Genetic testing can pinpoint rare genetic disorders or chromosomal abnormalities contributing to male infertility. Similarly, microbial analysis can enhance personalized medicine approaches by identifying specific dysbiotic patterns or microbial biomarkers associated with infertility [137,138,139].

Future research avenues should encompass extended clinical trials for microbial-based therapies, a deeper understanding of the interplay between microbiota, host factors, and reproductive health, and leveraging technological advancements like high-throughput sequencing and omics methodologies. This will provide a more comprehensive insight into the molecular mechanisms underpinning male infertility [118,119,121]. The development of innovative delivery mechanisms, such as nanoparticles or gene therapy techniques, can further enhance the efficacy and precision of microbial-based interventions [140,141,142]. Thus, it is imperative for upcoming research to not only deepen the understanding of the genital microbiota’s role in male infertility but also craft more precise therapeutic strategies, all while addressing the ethical and safety concerns tied to microbial-based therapies.

## 8. Conclusions

Grasping the impact of microbial factors on male infertility is essential for both accurate diagnosis and effective management of this intricate condition. Male infertility has been identified to have a strong correlation with microbial dysbiosis in the genital microbiota, which can directly compromise sperm health and overall fertility potential. Recent strides in microbiome research and microbial analysis techniques, such as next-generation sequencing, have shed light on the underlying causes of infertility, revealing potential etiologies behind cases previously deemed idiopathic. These insights have paved the way for the development of innovative therapeutic strategies, including microbiome therapy, aiming the reestablish a harmonious microbiome.

Disequilibrium in the human microbiome can manifest as a spectrum of diseases, many of which may benefit from interventions that aim to restore a balanced microbiome. This understanding has catalyzed the emergence of potential therapeutic modalities, including targeted antimicrobial therapies, probiotics, prebiotics, and fecal microbiota transplantation. These interventions hold promise in enhancing fertility outcomes. The increasing evidence underscoring the relationship between the human microbiome and diverse diseases has positioned it at the forefront of medical research, offering potential avenues for disease prevention, management, and prediction.

By delving deeper into the microbiome of infertile males, a holistic understanding of the etiology of male infertility can be achieved. Such knowledge is pivotal in designing microbiome-centric interventions tailored to address the unique fertility challenges faced by infertile males. Merging these interventions with personalized medicine strategies heralds a promising trajectory for addressing male infertility. As the understanding of the microbiome’s influence on male infertility deepens, the adoption of precision medicine—crafting treatments based on individual genetic, environmental, and microbial factors—emerges as a cornerstone, promising a nuanced and effective approach to this multifaceted condition.

Nevertheless, while these perspectives are promising, there remains an urgent need for further research to validate and refine these methodologies for their broader clinical application in addressing male infertility. Anticipated future research endeavors will further elucidate the role of microbial factors in male infertility, laying the foundation for the evolution of more potent treatment modalities.

## Data Availability

Not applicable.
